# The Association Between Autism Spectrum Traits and Age-Related Spatial Working Memory Decline: A Large-Scale Longitudinal Study

**DOI:** 10.1093/geront/gnaf096

**Published:** 2025-03-12

**Authors:** Saloni Ghai, Aphrodite Eshetu, Anne Corbett, Clive Ballard, Dag Aarsland, Adam Hampshire, Elizabeth O’Nions, William Mandy, Joshua Stott, Gavin R Stewart, Amber John

**Affiliations:** Research Department of Clinical, Educational and Health Psychology, University College London, London, UK; Research Department of Clinical, Educational and Health Psychology, University College London, London, UK; Medical School, University of Exeter, Exeter, UK; Medical School, University of Exeter, Exeter, UK; Old Age Psychiatry, King’s College London, London, UK; Faculty of Medicine, Imperial College London, London, UK; Department of Neuroimaging, King's College London, London, UK; Research Department of Clinical, Educational and Health Psychology, University College London, London, UK; Research Department of Clinical, Educational and Health Psychology, University College London, London, UK; Research Department of Clinical, Educational and Health Psychology, University College London, London, UK; Research Department of Clinical, Educational and Health Psychology, University College London, London, UK; Social Genetic and Developmental Psychiatry Centre, King’s College London, London, UK; Research Department of Clinical, Educational and Health Psychology, University College London, London, UK

**Keywords:** Autism, Cognitive decline, Trajectory analysis, Working memory

## Abstract

**Background and Objectives:**

Based on mixed findings from previous research, researchers have hypothesized autism may be a protective or risk factor for age-related cognitive decline/dementia, or that autism does not influence it (parallel aging). To differentiate between hypotheses, longitudinal studies that account for autism underdiagnosis, are needed and lacking. This study examined if higher autistic traits in adults aged 50+ are associated with a greater risk of spatial working memory (SWM) decline, a key cognitive domain affected in both healthy aging and autism.

**Research Design and Methods:**

Participants from the online PROTECT cohort (*n* = 13,390) were classified into 3 groups based on autism spectrum traits (AST): high (H-AST, *n* = 205), intermediate (I-AST, *n* = 589), and no traits (COA, *n* = 12,451). Spatial working memory performance was captured annually across 7 years. Growth mixture models (GMM) and latent growth curve models were estimated to examine the relationship between AST and SWM.

**Results:**

Growth mixture models revealed an optimal 1-class quadratic solution, consistent across groups. There were no significant differences between AST groups in baseline SWM (*p* = .837). Autism spectrum traits were not associated with SWM at baseline (*B* = 0.01, *SE* = 0.05, *p* = .901) or SWM trajectory (*B* = 0.00, *SE* = 0.01, *p* = .856), regardless of accounting for covariates.

**Discussion and Implications:**

Findings suggest a single SWM trajectory in middle-aged/older adults with higher autistic traits and no autistic traits. Future research should address if these broader autism phenotype results are replicated in diagnosed autism groups.

Autism is a lifelong set of highly heritable heterogeneous neurodevelopmental conditions characterized by differences in social communication and repetitive patterns of sensory-motor behaviors (American Psychiatric Association, 2013). Recent estimates suggest that Autism has a global prevalence of ~1% (Santomauro et al., 2024). However, due to historical changes to the diagnostic criteria for autism (e.g., from a narrow to wide diagnostic criteria, historically being a predominately male diagnosis), many people remain undiagnosed (Happé & Frith, 2020; Lai & Baren Cohen, 2015). For example, a study using UK healthcare records found that only 1 in 18,000 adults over the age of 50 had an autism diagnosis in 2018, suggesting that only 1 in 9 autistic people in this age group are diagnosed (O’Nions et al., 2023). Additionally, due to these changes in diagnostic criteria and the high rates of underdiagnosis in older aged populations, we know little about the needs of autistic people as they age (Mason et al, 2022).

Autism is often viewed as being part of a spectrum, where it exists at the end of a continuum of high to low autistic traits found in the general population (Constantino and Todd, 2003; Hoekstra et al., 2007). Additionally, these traits are found to have strong genetic overlap with diagnosed autism (Bralten et al., 2018). Furthermore, classifying autistic traits as natural human variations existing on a continuum, as opposed to medical deficits, also aligns with the neurodiversity perspective which returns autonomy to the autistic community regarding their care and acknowledges not only differences but also strengths associated with autism (Kapp et al., 2013). As such, using a dimensional trait-based approach to study Autism has become increasingly common, particularly in historically overlooked populations, for example, women/girls, older people. The effectiveness of this approach is also strengthened by growing evidence that individuals with high autistic traits are often found to have similar social, health, and cognitive profiles to diagnosed autistic samples (Stewart et al., 2020, 2021, 2023). Taken together, studying autistic traits in older age is a convenient way to bridge 2 high-priority public health issues—understanding the needs of neurodivergent people, and understanding the needs of older people.

A central issue in autism and aging research is whether older autistic and high autistic trait people are at risk of accelerated cognitive aging. Cross-sectional evidence has indicated that older autistic people often self-report that their cognitive abilities are declining (Klein et al., 2023; Stewart et al., 2018, 2024), but there is little cross-sectional evidence about objective cognitive performance changes. Cognitive decline is a key issue for society given the growing prevalence of dementia (Nichols et al., 2022), which is a WHO public health priority. Factors implicated in age-related cognitive decline, such as a decrease in interference inhibition (Earles et al., 1997), information processing speed (Caplan and Waters, 2005), social participation (Lövdén et al., 2005), and an increase in depressive symptoms (Paterniti et al., 2002), are all elevated in autistic populations (Haigh et al., 2018; Hossain et al., 2020; Stewart et al., 2024; Tonizzi et al., 2022). Thus, understanding whether there are differences in how particular domains of cognition might change with age in autistic and high autistic trait groups compared to non-autistic and low autistic trait groups is a topic of great interest. Geurts and Vissers (2012) have proposed 3 hypotheses of how cognition may change in autistic populations: that autistic individuals show similar age-related changes to non-autistic people, that is parallel development, that autism may have a detrimental effect on age-related cognitive changes, that is, steeper decline (double jeopardy hypothesis), or that autism may have a protective effect on age-related cognitive changes (safeguard hypothesis). Furthermore, these patterns of change may be domain-specific.

Working memory (WM) is a particular area of interest in this regard since working memory differences have been widely documented across early life through to middle age and later life in autistic populations (Kercood et al., 2014; Steele et al., 2007; Stewart et al., 2018, 2023). While some cross-sectional studies comparing WM between older and younger autistic adults have suggested either a parallel (Geurts and Vissers, 2012) or safeguarding effect (Lever et al., 2015) of autism on WM decline, these studies are limited as findings could be due to differences in groups. To properly test the 3 hypotheses relating autism to changes in WM with age, longitudinal within-group studies are needed and the current study is the first (to our knowledge) to do this. Consequently, this study aimed to investigate if higher autism spectrum traits (AST) in middle-aged/older adults predicted memberships of different trajectories of age-related changes in spatial working memory (SWM) compared with no AST. It was hypothesized that middle-aged/older adults would show age-related SWM decline independent of AST, but trajectories of age-related change may differ in those with higher AST compared with no AST.

## Method

### Design and Procedure

This study used data from PROTECT (https://www.protectstudy.org.uk), a U.K.-based longitudinal cohort study that commenced in 2014. Participants were recruited through national and local publicity (including radio and print media), the Joint Dementia Research service, and existing study cohorts.

After online registration, participants completed baseline online cognitive testing and cognitive and behavioral questionnaires, with annual follow-up requests. Data for the present study consisted of participants who registered with PROTECT from 2014 until 2020 and who completed cognitive testing for at least 3 years (including baseline), to allow for longitudinal modeling (Curran et al., 2010). Ethical approval for the PROTECT study was granted by the UK London Bridge National Research Ethics Committee (Ref: 13/LO/1578).

### Participants

Participants (*N* = 13,390) were people aged 50+ without dementia who had a good understanding of English, ability to provide consent to participate, and the ability to use a computer with internet access.

### Measures

#### Key Study Variables

##### Autism Spectrum Traits

Participants were allocated into 1 of 3 AST groupings using the PROTECT AST screener questions, which comprised of 5 yes/no items focusing on childhood (*n* = 2; e.g., knowing how to get along with other children) and current (*n* = 3; e.g., understanding others’ perspectives) sociocommunicative autism-related traits. Participants with a self-reported autism diagnosis (*n* = 22) and those who endorsed both childhood traits and at least 2 current traits formed the High AST (H-AST, *n* = 205) group (i.e., scores = 4–5), which represented a probable autism group. The Intermediate AST (I-AST, *n* = 589) group represented the broader autism phenotype group and consisted of participants who endorsed either current or childhood sociocommunicative autistic traits (i.e., scores = 1–4) but did not meet threshold for probable autism. The Comparison Adults (COA, *n* = 12,451) group served as a no-autistic traits comparison group (scores = 0). This screener has shown good internal consistency (Cronbach’s *α* = .82), sensitivity (82%), and specificity (94%) for identifying individuals with an autism diagnosis (Stewart et al., 2020).

##### Spatial Working Memory

Cognitive testing in PROTECT was conducted using the validated PROTECT Cognitive Test System (PCTS; Corbett et al., 2015) and the validated CogTrack system (Wesnes et al., 2017; see Supplementary Material for information on the full cognitive battery).

Spatial working memory was measured through the PCTS, using an adapted version of the validated CANTAB Self-Ordered Search task (Owen et al., 1990) involving searching in on-screen boxes to find a target symbol (Supplementary Material). The main outcome measure was the task summary score (henceforth SWM score), with a higher score indicating more efficient SWM maintenance with no limit on the maximum score. The task has successfully been used to assess SWM in autism (Steele et al., 2007). To exclude any effect of hardware issues or participants not understanding instructions on scores, any scores of 0 were treated as missing data.

#### Covariates and Sample Characteristics

Participants self-reported their gender, marital status, education level, ethnicity, employment status, and physical and psychiatric diagnoses (including an autism diagnosis) at baseline using PROTECT’s online survey platform.

Subjective cognitive decline (SCD) was measured using the self-report version of the validated 16-item Informant Questionnaire on Cognitive Decline in the Elderly Self (IQCODE-SF) (Jorm,1994, 2004). Participants indicate whether they have improved (= 1), stayed the same (= 3), or become worse (= 5) at learning new things, remembering important dates, etc. A mean score of ≥ 3.31 suggests a person has experienced cognitive decline over the past 10 years.

##### Covariates

Age, gender, sustained attention, education level, anxiety, and depression were included in latent growth curve models (LGCM). Anxiety and depression were accounted for as participants may have scored highly on the AST screener due to sociocommunicative difficulties experienced as a result of mental health problems, rather than autism.

Sustained Attention was measured in CogTrack using the Digit Vigilance Task (Lin et al., 2018). The main outcome was performance accuracy at baseline, which indexed participants’ sustained attention capacity that was accounted for to ensure any differences in SWM scores were not due to lack of sustained attention during testing.

Recent symptoms of depression were measured using the 9-item self-report Patient Health Questionnaire (Kroenke et al., 2001). Participants indicated how bothered they have been over the past 2 weeks by problems relating to the 9 DSM-IV criteria for depression using a 4-point Likert scale ranging from 0 = “not at all” to 3 = “nearly every day” (Maximum score = 27).

Recent symptoms of anxiety were measured using the 7-item Generalized Anxiety Disorder self-report questionnaire (Spitzer et al., 2006). Participants rated how bothered they have been over the last 2 weeks by symptoms of anxiety using a 4-point Likert scale ranging from 0 = “not at all” to 3 = “nearly every day” (Maximum score = 21).

### Statistical Analysis

Growth mixture models (GMM) and latent growth curve models were estimated using Mplus version 8.8. 1-5 class GMMs were conducted using the total sample size (*N* = 13,390) to examine heterogeneity in the trajectory of SWM scores over time and determine the optimal number of latent classes. A 1-class GMM solution was first estimated to determine if a linear or quadratic slope best fit the trajectory of SWM scores. Factor loadings of time points (years of follow-up testing) were set to 0, 1, 2, etc., as the interval between time points was equal. Fit indices used to select the optimal class solution included the Akaike, Bayesian, and sample-size adjusted Bayesian information criterion indices, entropy values (measuring classification quality), the Vuong-Lo-Mendell-Rubin likelihood ratio test (LMR LRT), and the bootstrap likelihood ratio test (BLRT). The optimal model would have lower values on information criterion indices, higher entropy, and significant *p* values (*p* < .05) for the LMR LRT and BLRT. Fit indices, class sizes (classes should not represent less than 5% of the sample), and theoretical sensibility guided optimal model selection (Infurna and Grimm, 2018). GMMs were estimated with 200 initial stage random starts and 5 final stage optimizations to ensure the best log-likelihood value was replicated. Five bootstrap draws were used in the BLRT.

Latent growth curve models were estimated to examine how AST grouping and study covariates were associated with SWM scores at baseline (intercept) and SWM trajectory (slope). Standardized *β* for the association between each model predictor and SWM score was used as a measure of the magnitude of effect (Cohen, 1988). Conditional LGCMs were first estimated for each covariate, with a final model including all covariates. All covariates were specified as time-invariant. Goodness of fit was evaluated using the comparative fit index (>0.90), root mean square error of approximation index (<0.06), standardized root mean square residual (<0.08), and Tucker–Lewis index (>0.90) (Hu and Bentler, 1999; Smith and Ehlers, 2020). For both LGCMs and GMMs, the full information maximum likelihood method was used to estimate missing data to avoid bias from attrition and missing data.

## Results

### Descriptive Statistics

The proportion of females (*N* = 10,060) to males (*N* = 3,301) was 75.3%:24.7%. Median sample age was 62 years (IQR = 67 – 57) and was lower in the H-AST group than the COA (*p* = .027) and I-AST (*p* = .015) groups by ~2 years. Key demographic information across AST groups is presented in Table 1. Please refer to Supplementary Material for a summary of self-reported diagnoses across AST groups.

**Table 1.** Summary of Demographic Characteristics Across AST Groups

Demographic characteristic	Descriptor	COA (Max *n* = 12,451)	I-AST (Max *N* = 589)	H-AST (Max *N* = 205)	Group difference	Posthoc comparisons between AST groups reaching significance (*p* < .05)
Age (years)	Median (IQR) Range	62 (50–100) 57–67	62 (50–92) 57–67	60 (50–82) 56–66	*Χ* ^2^(2, 13235) = 6.78, *p* = .034^*^ η^2^ (*H*) = 0.00	*COA vs. H-AST: p = .027* ^*^ *I-AST vs. H-AST: p = .015* ^*^
Gender	Male (%) Female (%)	2,996 (24.1%) 9,447 (75.9%)	223 (37.9%) 366 (62. 1%)	58 (28.3%) 147 (71.7%)	*χ* ^2^ (1, 13237) = 58.75, *p* < .001^***^ φ_*c*_ = 0.07	COA vs. I-AST: p < .001*** for difference in % of males and females.
Ethnicity	White (%) Non-white (%)	12,261 (98.5%) 182 (1.5%)	570 (96.8%) 19 (3.2%)	200 (97.6%) 5 (2.4%)	*χ* ^2^ (1, 13237) = 12.47, *p* = .002^**^ φ_*c*_ = 0.03	COA vs. I-AST: p = .001*** for difference in % white ethnicity only
Marital status	Married Widowed Separated Divorced Cohabiting Single Civil partnership	8,506 (68.3%) 810 (6.5%) 196 (1.6%) 1,341 (10.8%) 781 (6.3%) 744 (6.0%) 65 (0.5%)	348 (59.1%) 36 (6.1%) 12 (2.0%) 78 (13.2%) 48 (8.2%) 64 (10.9%) 3 (0.5%)	133 (64.9%) 8 (3.9%) 3 (1.4%) 27 (13.2%) 11 (5.4%) 23 (11.2%) –	*χ* ^2^ (2, 13237) = 48.50, *p* < .001^***^ φ_*c*_ = 0.04	COA vs. I-AST: p < .001*** for difference in proportion of married participants
Education level	School to 16 School to 18 Diploma Undergraduate Postgraduate Doctorate	1,669 (13.6%) 1,402(11.3%) 2,466 (19.8%) 4,263 (34.3%) 2,143 (17.2%) 470 (3.8%)	67 (11.4%) 70 (11.9%) 106 (18.0%) 201 (34.1%) 116 (19.7%) 29 (4.9%)	25 (12.2%) 20 (9.8%) 36 (17.6%) 78 (38.0%) 38 (18.5%) 8 (3.9%)	*χ*2 (2, 13237) = 9.41, *p* = .494 *φ*_*c*_ = 0.02	
Employment status	Employed (%) Retired (%) Unemployed (%)	5,625 (45.2%) 330 (2.7%) 6,847 (52.1%)	275 (46.7%) 19 (3.2%) 295 (50.1%)	100 (48.8%) 13 (6.3%) 92 (44.9%)	*χ* ^2^ (2, 13236) = 13.85, *p* = .008^*^ φ_*c*_ = 0.02	*p > .05 for difference in % of employed, retired and unemployed participants between all groups.*

*Notes*: AST = autism spectrum traits; COA = comparison adults group; H-AST = high AST group; I-AST = intermediate AST group; IQR = interquartile range; *N* = number.

^*^ *p* < .05.

^**^ *p* < .01.

^***^ *p* < .001.

Chi-square tests of association revealed significant but small (*φ*_*c*_ = 0.07–0.2) or very small (*φ*_*c*_ < 0.07) differences between AST groups in gender, ethnicity, employment status, marital status (Table 1) and in participants meeting cutoff for SCD, anxiety, and depression (Table 2). Mixed ANOVA analyses revealed significant but very small differences in baseline age, depression, anxiety, sustained attention, and SCD (*η*^2^(*H*) ≤ 0.1). There were no significant group differences in baseline SWM scores (*p* = .837) (Table 2). All post hoc analyses were Bonferroni corrected for multiple comparisons.

**Table 2.** Sustained Attention, Spatial Working Memory, Depression, Anxiety and Subjective Cognitive Decline Scores at Baseline Across AST Groups

Measurement	Descriptor	COA (max *n* = 12,451)	I-AST (max *N* = 589)	H-AST (max *N* = 205)	Group difference	Posthoc comparisons between AST groups reaching significance (*p* < .05)
Sustained attention (digit vigilance accuracy rating in %)	Mdn (IQR) Range	100 (100 – 97.78) 0–100	97.78 (100–95.56) 24.44–100	100 (100 – 97.78) 80–100	*Χ* ^2^(2, 9244) = 10.28, *p* = .006^**^ η^2^ (H) = 0.00	*COA vs. I-AST: p = .003* ^**^
Spatial working memory (SOS task score)	Mdn (IQR) Range	8 (9-7) 3–44	8 (9-7) 4–17	8 (9-7) 4–20	*Χ* ^2^(2, 12238) = 0.36, *p* = .837 *η*^2^ (*H*) = 0.00	
Subjective cognitive decline (IQCODE-SF, max. score = 5, cut-off ≥ 3.31)	Mdn (IQR) Range	3.06 (3.19-3) 1–4.56	3.19 (3.31-3) 2.13–4.50	3.13 (3.31-3) 1.69–4.56	*Χ* ^2^(2, 13205) = 70.33, *p* < .001^***^ η^2^ (*H*) = 0.01	*COA vs. H-AST: p < .001* ^***^ *COA vs. I-AST: p < .001* ^***^
	N (% over cut-off)	1,796 (14.5%)	156 (26.5%)	56 (27.5%)	*Χ* ^2^ (2, 13205) = 87.06 *p* = < .001^***^ φ_*c*_ = 0.08	*COA vs. I-AST: p < .001* ^***^ *COA vs. H-AST: p = .007* ^**^
Depression (PHQ-9, max. score = 27, cut-off ≥ 10)	Mdn (IQR) Range	2 (3-0) 0–25	3 (6-1) 0–26	5 (8-2) 0–24	*Χ* ^2^(2, 13010^)^ = 275.88, *p* < .001^***^ η^2^ (*H*) = 0.02	*COA vs. H-AST: p < .001* ^***^ *COA vs. I-AST: p < .001* ^***^ *I-AST vs. H-AST: p < .001* ^***^
	*N* (% over cut-off)	607 (4.88%)	76 (12.90%)	40 (19.51%)	*Χ* ^2^(2, 13010^)^ = 149.92, *p* < .001***, *φ*_*c*_ = 0.11	*COA vs. I-AST: p = .005* ^**^ *COA vs. H-AST: p < .001* ^***^
Anxiety (GAD-7, max. score = 21, cut-off ≥ 10)	Mdn (IQR) Range	0 (2-0) 0–21	1 (4-0) 0–21	3 (5.5-0) 0–21	*Χ* ^2^(2, 13131^)^ = 259.00, *p* < .001^***^ η^2^ (*H*) = 0.02	*COA vs. H-AST: p = .007* ^**^
	*N* (% over cut-off)	303 (2.43%)	36 (6.11%)	25 (12.20%)	*Χ* ^2^(2, 13131^)^ = 98.00, *p* < .001***, *φ*_*c*_* = 0.09*	*COA vs. H-AST: p = .002* ^**^

*Notes*: AST = autism spectrum traits; COA = comparison adults group; GAD = Generalized Anxiety Disorder questionnaire; H-AST = high AST group; I-AST = intermediate AST group; IQR = interquartile range; IQCODE-SF = Informant Questionnaire on Cognitive Decline in the Elderly Self; *N* = number; PHQ = Patient Health Questionnaire; SOS Task = Self-Ordered Search Task.

^*^ *p* < .05.

^**^ *p* < .01.

^***^ *p* < .001.

### Growth Mixture Models

Fit indices used to determine if 1-class linear or 1-class quadratic GMM solution best fit the trajectory of SWM scores indicated a 1-class quadratic solution to best fit the data (Table 3). Although the Akaike, Bayesian, and sample-size adjusted Bayesian were lower for the 2-class quadratic solution and the LMR LRT and BLRT for the 2-class solution met significance (*p* < .05), the second class represented less than 5% of the data (*n* = 13) limiting its clinical significance.

**Table 3.** Class Sizes, Number of Free Parameters, Fit Indices and Entropy for Estimated 1–5 Class Growth Mixture Models

GMM (quadratic)	Class sizes	Free parameters (N)	BIC	SSBIC	AIC	BLRT	LMR LRT	Entropy
1-Class linear	13,390	16	240,792	240,754	240,702	-	-	-
1-Class quadratic	13,390	16	240,341	240,290	240,221	-	-	-
2-Class quadratic	13,377; 13	20	239,122	239,059	238,972	*p* < .001^***^	*p* = .013^*^	1.00
3-Classquadratic	144; 13; 13,233	24	238,847	238,771	238,667	*p* < .001^***^	*p* = .127	0.980
4-Class quadratic	135; 9; 6; 13,240	28	238,727	238,638	238,517	*p* < .001^***^	*p* = .055	0.985
5-Class quadratic	134; 274; 6; 9; 12,967	32	238,623	238,521	238,383	*p* < .001^***^	*p* = .045^*^	0.955

*Notes:* AIC = Akaike Information Criterion; BIC = Bayesian Information Criterion; BLRT = Bootstrap Likelihood Ratio Test; GMM = growth mixture model; LMR LRT = Vuong-Lo-Mendell-Rubin likelihood ratio test (LMR LRT); *N* = number; SSBIC = Sample-size Adjusted Bayesian Information Criterion.

^a^Class counts for latent classes based on the estimated model, AIC, BIC, and SSBIC, are rounded to the nearest whole number.

^*^ *p* < .05.

^**^ *p* < .01.

^***^ *p* < .001.

**Figure 1.**
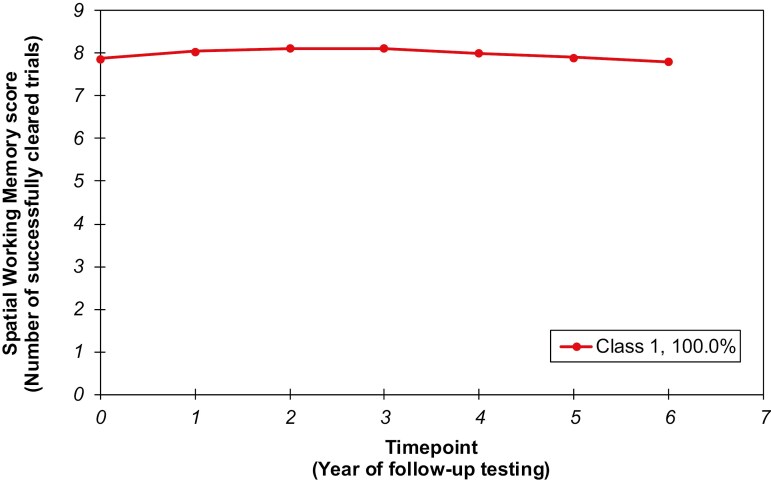
Median SWM scores from baseline to 6 years of follow-up testing estimated for a 1-class quadratic growth mixture model solution. The trajectory of median SWM scores (estimated for a 1-class quadratic GMM solution) from baseline to 6 years of follow-up testing. The model suggests stability in typical SWM scores from baseline until time-point 6, with 50% of the sample clearing ≥ 8 trials of the task at all time-points. GMM = growth mixture model; SWM = spatial working memory.

Figure 1 and estimated median SWM scores across the 7 years of testing (Supplementary Material) illustrated stability in the trajectory of SWM scores, with median SWM score remaining ~8 trials cleared throughout the 7 years. There was high variance in baseline SWM scores (*Var* = 1.65, *SE* = 0.06, *p* < .001) and small variance in the linear (*Var* = 0.09, *SE* = 0.02, *p* < .001) and quadratic terms for SWM trajectory (*Var* = 0.00, *SE* = 0.00, *p* < .001).

### Latent Growth Curve Models

Univariate LGCMs indicated that age, depression, anxiety, sustained attention, education level, and gender showed weak (0.1 ≤ *β* ≤ 0.3) but significant associations with SWM scores at baseline (Supplementary Material). Autism spectrum traits were not associated with SWM at baseline (*B* = 0.01, *SE* = 0.05, *p* = .901) or SWM trajectory (*B* = 0.00, *SE* = 0.01, *p* = .856), regardless of accounting for covariates (Supplementary Material).

In the multivariate LGCM, depression, anxiety, sustained attention, and education level continued to show weak associations with baseline SWM, but gender and age were now moderately associated with baseline SWM (0.3 ≤ *β* ≤ 0.5). Neither AST nor any covariates were significantly associated with SWM trajectory (*p* > .05).

Results for all LGCMs demonstrated a good model fit based on the root mean square error of approximation index, Tucker–Lewis index, comparative fit index, and standardized root mean square residual model fit indices, indicating good correspondence between the fitted models with observed data (Supplementary Material).

## Discussion

We investigated whether higher autistic traits were associated with different trajectories of age-related SWM decline in midlife/older adulthood. The main finding of this study is that a single trajectory of SWM development is likely in middle-aged/older adults with high autistic traits and middle-aged/older adults with no autistic traits, providing evidence against a double jeopardy or safeguarding hypotheses in this cognitive domain.

This is the first longitudinal study to examine autistic traits and SWM over a period of 7 years, and our study with a large sample builds on previous smaller-scale cross-sectional studies, some of which have also found parallel trajectories in particular cognitive domains (Geurts and Vissers, 2012). Importantly, in this large trait-based sample, we found a single trajectory of SWM development in middle-aged/older adults with varying levels of autistic traits. This study comprises a larger cohort than in previous studies (Geurts and Vissers, 2012; Torenvliet et al., 2022), thus making it less likely that the non-significant findings in this area are due to low power.

High variance in baseline SWM scores and significant but small variance in the trajectory of SWM scores suggested considerable heterogeneity in baseline SWM but a similar SWM trajectory between participants. However, our autistic trait groupings were not associated with differences in SWM scores at baseline or SWM trajectory. Thus, cross-sectional findings of SWM differences between autistic children/younger adults and non-autistic groups (Luna et al., 2007; Steele et al., 2007) were not replicated in the present study. It is possible that the SWM gap between adults with high and low autistic traits decreases at some point in young and middle adulthood prior to the age range examined in this study. However, results suggest a common SWM trajectory in mid-older adulthood.

Another finding of this study is that large-scale cumulative age-related changes in SWM were not observed across the 6 years of follow-up testing and age was not associated with SWM trajectory, which is somewhat unexpected. There are several possibilities for this, firstly the PROTECT cohort is mainly cognitively healthy and drop out may be influenced by cognitive decline, thus there could be a survivor effect with participants who continued to participate across the 7 years (Golomb et al., 2012). A higher cognitive reserve in comparison to middle-aged/older adults in the wider population may prevent steeper age-related cognitive changes by enabling the adoption of new strategies to compensate for initial difficulties with performance (Giogkaraki et al., 2013), Secondly the mean sample age was 60 years, and age-related cognitive changes might have possibly been more pronounced at a later stage. Additionally, stability in scores across time may be due to practice gains on performance (Brehmer et al., 2012), something which has been observed in previous longitudinal work (Goh et al., 2012).

### Strengths and Limitations

A strength of PROTECT is its use of online recruitment and cognitive testing that allows for large-scale recruitment and testing of participants without the need for in-person assessments. However, the sample may not be representative of the entire population of middle-aged/older adults in the UK as it is limited to those who can confidently use a computer and access the internet. Additionally, the autistic trait screener correlates with and has good cut-off overlap with widely used measures of autistic traits (AQ-10, RAADS-14) (Stewart et al., 2020). However, as items solely focus on sociocommunicative difficulties in autism and not restrictive/repetitive behaviors or sensory problems, participants may have scored high on the screener due to sociocommunicative difficulties independent of autistic traits. Hence, depression and anxiety were accounted for in LGCMs examining the association between autistic traits and SWM.

The intermediate and high autistic trait groups were not limited to a clinical autism diagnosis, which helps account for high rates of autism underdiagnosis in middle-aged/older adults and incorporated the broader autism phenotype group. However, this approach should be complemented by studies with robust clinical diagnoses. As demographic questionnaires did not consider intellectual disability (ID), group differences in SWM associated with ID were not accounted for. This makes it difficult to generalize results to those with high autistic traits and co-occurring ID, which is negatively associated with WM and visuospatial problem-solving abilities (Numminen et al., 2001) and is one of the most common comorbidities in autism (Matson and Goldin, 2013). Lastly, the present sample was of predominantly white ethnicity (98.4%), which does not completely reflect the U.K. population aged 50+ (93.6% White, Office for National Statistics, 2022).

## Implications and Future Research

Despite some limitations in the generalizability of findings, results provide important and novel insight into the association between autistic traits and SWM trajectory in middle-aged/older adults, and despite our null results, provide evidence that autistic/high autistic trait people in midlife and older age may not be at risk of accelerated decline in their spatial working memory ability.

When extending the findings of this study in future longitudinal work, it would be useful to conduct follow-up testing over a longer period to ensure gradual age-related changes in SWM are captured. This will also help interpret the association between autistic traits and SWM trajectory with greater certainty. Furthermore, incorporating a wider age span from young to older adulthood will help determine if a single SWM trajectory is present across the adult lifespan regardless of autistic traits, or if autistic traits may have a protective effect on age-related SWM decline in young-middle adulthood.

Our findings are important for diagnosed and undiagnosed autism groups concerned about future risk of dementia. In future studies, if a common SWM trajectory is replicated with further longitudinal testing, it would be beneficial to test if middle-aged/older adults with varying levels of autistic traits can benefit from WM training interventions targeting SWM functioning that have proven to be effective in older adults without autism (Brehmer et al., 2012).

## Conclusion

This study aimed to investigate if higher autistic traits in middle-aged/older adults are associated with different trajectories of age-related spatial working memory decline compared to middle-aged/older adults with low autistic traits. A single trajectory emerged, with stability in spatial working memory performance across the 7 years of testing. Autistic traits were not associated with baseline performance or trajectory, suggesting that autistic traits do not confer risk to an accelerated decline in spatial working memory in mid-older adulthood. Considering established differences in spatial working memory between autistic/high-trait and non-autistic/low-trait children and younger adults, no performance differences were found at baseline between the high and no autistic traits groups. These findings suggest high autistic traits may have a protective effect on age-related working memory decline in early and mid-adulthood, although further longitudinal work is needed to explore this.

## Supplementary Material

gnaf096_suppl_Supplementary_Materials

## Data Availability

Data, analytic methods or materials for this study are not available to other researchers for replication purposes due to ethics requirements and data access agreements. If data are available, where they can be accessed—not applicable. The study reported in the manuscript was not preregistered.
