# Supplementary material for: The Association Between Autism Spectrum Traits and Age-Related Spatial Working Memory Decline: A Large-Scale Longitudinal Study
Source: Gerontologist. 2025 Mar 12;65(5):gnaf096. doi: 10.1093/geront/gnaf096 (PMC12036657; doi:10.1093/geront/gnaf096)
Supplement: gnaf096_suppl_Supplementary_Materials [file gnaf096_suppl_supplementary_materials.docx]

# **Supplementary Materials**

# **Supplementary Material for The Association between Autism Spectrum Traits and Age-Related Spatial Working Memory Decline: A Population-based Cohort Study**

# Contents

1. PROTECT Cognitive Testing Battery.
2. Self-reported Psychiatric Diagnoses across AST Groups
3. Estimated Median and Mean SWM Scores at each Time-Point for the Optimal Quadratic One-class GMM
4. Conditional Latent Growth Curve Model Estimates and Fit Indices

**Description of Cognitive Tasks**

Participants completed cognitive tasks within two blocks; the PROTECT package, and the CogTrack package.

**Assessment of cognition via the PROTECT package (Corbett et al., 2015)**

The PROTECT package of cognitive tasks are based on tests which have been used widely over the last 30 years in a wide range of clinical trials with young and elderly individuals, including those with pathological age-related cognitive declines.

The instructions are presented visually at the start of each testing session and also at the start of each task.

The following tests were self-administered in the below order:

1. *Verbal Reasoning –* measured using an adapted version of the Baddeley Grammatical Reasoning. This test correlates with measures of general intelligence and involves determining the accuracy of a series of grammatical statements about a picture. The outcome measure was the total number of trials answered correctly in 90 seconds, minus the number answered incorrectly, presented as a total score.
2. *Spatial Working Memory –* measured through the Self-Ordered Search test. Participants searched a series of on-screen boxes to find a hidden symbol. Once found, participants searched for a new symbol, remembering that a symbol would never be hidden in the same box twice. The main outcome was the average number of boxes in the successfully completed trials.
3. *Verbal working memory –* measured through the digit span task, which has been widely cited in the neuropsychological literature and used in many commercially available brain training devices. The test used a ratchet style approach in which each successful trial is followed by a new sequence that is 1 digit longer than the last and each unsuccessful trial is followed by a new sequence that is 1 digit shorter than the last. This allows an accurate estimate of digit span to be made quickly. The main outcome measure was the average number of digits in all successfully completed trial.
4. *Visual working memory –* measured through the paired associates learning test, widely used in assessment of cognitive deterioration in Alzheimer disease. Participants see a series of objects, one at a time, and select the correct location of each object in “windows” they had previously been shown. The version also used a ratchet style approach. The main outcome measure was the average number of correct object place associations (“paired associates”) in the trials that were successfully completed.

**Assessment of cognition via CogTrack (Wesnes et al., 2017)**

The CogTrack System is an online set of cognitive tasks (www.wesnes.com) based on tests which have been used widely over the last 30 years in a wide range of clinical trials with young and elderly individuals, including those with pathological age-related cognitive declines.

The instructions are presented visually at the start of each testing session and also at the start of each task. In task responses are made using the right arrow on the keyboard in two tasks, and the left and right arrows in the other two. The participants are instructed to rest their finger(s) lightly upon the key(s) throughout each task. The speed and accuracy of every response are recorded.

The following tests were self-administered in the below order:

1. *Cued Visual Episodic Secondary Memory Retrieval* – measured using a picture recognition test. A series of 20 pictures of everyday scenes and objects is presented on the screen, at the rate of one picture every 3s, for the participant to remember. The participant is instructed that the pictures will all be shown again later mixed with very similar ones. For each picture, the participant has to indicate whether or not it was the precise picture shown earlier, pressing the right arrow key on the keyboard if it was, and the left arrow if it was not; as quickly and accurately as possible. The main outcome measure was the accuracy rating (%) and the reaction time (msec).
2. *Attention –* measured using a Simple reaction time test. The participant is instructed to press the right arrow key on the keyboard as quickly as possible every time a right facing arrow containing the word ‘YES’ is presented in the centre of the screen. The participant is informed that only this stimulus will be presented and that it will remain there until a response is made. Fifty stimuli are presented with random interstimulus interval between one and 3.5s. The main outcome measure was the reaction time (msec).
3. *Sustained Attention and Information Processing* – measured using a digit vigilance test. A target digit from one to nine is randomly selected and constantly displayed to the righthand side of the screen. A series of 450 digits is then presented one at a time in the centre of the screen at the rate of 150 per minute. The participant is required to press the right arrow key on the keyboard as quickly as possible every time a presented digit matches the target digit on the right. The main outcome measure was the accuracy rating (%), the reaction time (msec), and the false alarm score.
4. *Attention and Information Processing* – measured using a choice reaction time test. The two possible stimuli in this task are either the right facing arrow used in Simple Reaction Time task, or a left facing version of the arrow, with the word ‘NO’ in the middle. On each of 50 successive trials, one of these two stimuli is selected randomly (but with equal probability) presented in the centre of the screen, remaining there until a response is made. The interval between successive trials varies randomly between one and 3.5s. The participant is required to respond as quickly and accurately as possible. The main outcome measure was the accuracy rating (%) and the reaction time (msec).

**References:**

Corbett, A., Owen, A., Hampshire, A., Grahn, J., Stenton, R., Dajani, S., Burns, A., Howard, R., Williams, N., Williams, G., & Ballard, C. (2015). The Effect of an Online Cognitive Training Package in Healthy Older Adults: An Online Randomized Controlled Trial. *Journal of the American Medical Directors Association*, *16*(11), 990–997. https://doi.org/10.1016/j.jamda.2015.06.014

Wesnes, K. A., Brooker, H., Ballard, C., McCambridge, L., Stenton, R., & Corbett, A. (2017). Utility, reliability, sensitivity and validity of an online test system designed to monitor changes in cognitive function in clinical trials. *International Journal of Geriatric Psychiatry*, *32*(12), e83–e92. https://doi.org/10.1002/gps.4659

**Table S1**

*Self-reported Psychiatric Diagnoses across AST Groups*

| Diagnosis | COA | I-AST | H-ST |
| --- | --- | --- | --- |
| Mild Cognitive Impairment | 0.22% (N = 28) | 0.17% (N = 1) | 1.5% (N = 3) |
| Obsessive-compulsive disorder | 0.31% (N = 39) | 0.68% (N = 4) | 3.41% (N = 7) |
| Schizophrenia | 0.02% (N = 3) | 0.17% (N = 1) | 1.46% (N = 3) |
| ADHD | 0.05% (N = 6) | 1% (N = 1) | 5% (N = 5) |
| Current of Past Anxiety disorder (Panic disorder, social anxiety and/or generalized anxiety). | 16.51% (N = 2056) | 25.64% (N = 151) | 35.61% (N = 73) |

*Note. COA = Comparison adults group, I-AST = intermediate AST group, H-AST = high AST group, N = number.*

**Table S2**

*Estimated Median and Mean SWM Scores at each Time-Point for the Optimal Quadratic One-class GMM*

| Time-Point  (Years of Follow-up Testing) | Mdn | Percentiles  40%/80% | Mean | Skewness | Kurtosis | SD | Range | |
| --- | --- | --- | --- | --- | --- | --- | --- | --- |
| 0 | 8 | 7:9 | 7.88 | 1.64 | 22.89 | 2.04 | 3-44 |  |
| 1 | 8 | 8:10 | 8.12 | 0.18 | 0.89 | 1.83 | 4-20 |  |
| 2 | 8 | 8:10 | 8.12 | 0.31 | 1.10 | 1.78 | 4-20 |  |
| 3 | 8 | 8:10 | 8.12 | 0.36 | 1.47 | 1.78 | 4-20 |  |
| 4 | 8 | 8:9 | 8.01 | 0.49 | 1.66 | 1.72 | 4-20 |  |
| 5 | 8 | 7:9 | 7.90 | 0.29 | 0.57 | 1.61 | 4-18 |  |
| 6 | 8 | 7:9 | 7.90 | 0.24 | 0.44 | 1.62 | 4-18 |  |

*Note.* Mdn = median, SD = standard deviation

^a^40%/80% percentiles illustrate the middle 40% of the distribution of SWM scores at each time-point.

***Table S3***

*Conditional Latent Growth Curve Models Estimates and Fit Indices*

| Model Variables | B  (Intercept) | SE | B  (Slope) | SE | β  (Intercept) | SE | β  (Slope) | SE | RMSEA | TLI | CFI | SRMR |
| --- | --- | --- | --- | --- | --- | --- | --- | --- | --- | --- | --- | --- |
| Age | -0.05*** | 0.00 | 0.00 | 0.00 | -0.29*** | 0.01 | -0.05 | 0.03 | 0.037 | 0.964 | 0.964 | 0.024 |
| AST | 0.01 | 0.05 | 0.00 | 0.01 | 0.00 | 0.01 | 0.01 | 0.03 | 0.036 | 0.964 | 0.964 | 0.023 |
| AST  (Accounting for Anxiety and Depression) | 0.05 | 0.05 | 0.00 | 0.01 | 0.01 | 0.01 | 0.01 | 0.03 | 0.033 | 0.962 | 0.966 | 0.022 |
| Anxiety (GAD-7) | -0.03*** | 0.01 | 0.00 | 0.00 | -0.07*** | 0.01 | 0.00 | 0.03 | 0.037 | 0.964 | 0.964 | 0.024 |
| Depression (PHQ-9) | -0.02*** | 0.01 | 0.00 | 0.00 | -0.05*** | 0.01 | 0.00 | 0.03 | 0.037 | 0.963 | 0.963 | 0.025 |
| Sustained Attention (Digit Vigilance Accuracy in %) | 0.02*** | 0.01 | 0.00 | 0.00 | 0.10*** | 0.02 | 0.02 | 0.04 | 0.041 | 0.957 | 0.957 | 0.026 |
| Education Level | 0.10*** | 0.01 | 0.00 | 0.00 | 0.10*** | 0.01 | 0.01 | 0.03 | 0.036 | 0.964 | 0.964 | 0.023 |
| Gender | -0.39*** | 0.04 | 0.00 | 0.00 | -0.29*** | 0.03 | -0.05 | 0.08 | 0.036 | 0.964 | 0.964 | 0.023 |
| Conditional LGCM:  (1) Gender  (2) Depression  (3) Anxiety  (4) Age  (5) Education  (6) Digit Vigilance Accuracy (%) | -0.51***  -0.02*  -0.04***  -0.06***  0.07***  0.02*** | 0.04  0.01  0.01  0.01  0.01  0.00 | -0.01  0.00  0.00  0.00  0.00  0.00 | 0.01  0.00  0.00  0.00  0.00  0.00 | -0.38***  -0.03*  -0.06***  -0.32***  0.07***  0.08*** | 0.03  0.02  0.02  0.01  0.01  0.02 | -0.07  0.02  0.00  -0.02  0.00  0.02 | 0.09  0.05  0.05  0.04  0.04  0.04 | 0.032 | 0.957 | 0.964 | 0.021 |

*Note.* SE = standard error, RMSEA = root mean square error of approximation index, TLI = Tucker-Lewis index, CFI = comparative fit index, SRMR = standardised root mean square residual.

*p < .05, **p < .01, ***p < .001
